# OPC-167832, a Novel Carbostyril Derivative with Potent Antituberculosis Activity as a DprE1 Inhibitor

**DOI:** 10.1128/AAC.02020-19

**Published:** 2020-05-21

**Authors:** Norimitsu Hariguchi, Xiuhao Chen, Yohei Hayashi, Yoshikazu Kawano, Mamoru Fujiwara, Miki Matsuba, Hiroshi Shimizu, Yoshio Ohba, Izuru Nakamura, Ryuki Kitamoto, Toshio Shinohara, Yukitaka Uematsu, Shunpei Ishikawa, Motohiro Itotani, Yoshikazu Haraguchi, Isao Takemura, Makoto Matsumoto

**Affiliations:** a Infectious Diseases Unit, Department of Medical Innovations, New Drug Research Division, Otsuka Pharmaceutical Co., Ltd., Tokushima, Japan; b Medicinal Chemistry Research Laboratories, New Drug Research Division, Otsuka Pharmaceutical Co., Ltd., Tokushima, Japan; c Pharmaceutical Business Division, Otsuka Pharmaceutical Co., Ltd., Tokyo, Japan

**Keywords:** OPC-167832, DprE1 inhibitor, carbostyril derivative, antituberculosis agent

## Abstract

There is an urgent need for new, potent antituberculosis (anti-TB) drugs with novel mechanisms of action that can be included in new regimens to shorten the treatment period for TB. After screening a library of carbostyrils, we optimized 3,4-dihydrocarbostyril derivatives and identified OPC-167832 as having potent antituberculosis activity. The MICs of the compound for Mycobacterium tuberculosis ranged from 0.00024 to 0.002 μg/ml. It had bactericidal activity against both growing and intracellular bacilli, and the frequency of spontaneous resistance for M. tuberculosis H37Rv was less than 1.91 × 10^−7^. It did not show antagonistic effects with other anti-TB agents in an *in vitro* checkerboard assay. Whole-genome and targeted sequencing of isolates resistant to OPC-167832 identified decaprenylphosphoryl-β-d-ribose 2′-oxidase (DprE1), an essential enzyme for cell wall biosynthesis, as the target of the compound, and further studies demonstrated inhibition of DprE1 enzymatic activity by OPC-167832. In a mouse model of chronic TB, OPC-167832 showed potent bactericidal activities starting at a dose of 0.625 mg/kg of body weight. Further, it exhibited significant combination effects in 2-drug combinations with delamanid, bedaquiline, or levofloxacin. Finally, 3- or 4-drug regimens comprised of delamanid and OPC-167832 as the core along with bedaquiline, moxifloxacin, or linezolid showed efficacy in reducing the bacterial burden and preventing relapse superior to that of the standard treatment regimen. In summary, these results suggest that OPC-167832 is a novel and potent anti-TB agent, and regimens containing OPC-167832 and new or repurposed anti-TB drugs may have the potential to shorten the duration of treatment for TB.

## Abstract (precis)

There is an urgent need for new, potent antituberculosis (anti-TB) drugs with novel mechanisms of action that can be included in new regimens to shorten the treatment period for TB. After screening a library of carbostyrils, we optimized 3,4-dihydrocarbostyril derivatives and identified OPC-167832 as having potent antituberculosis activity. The MICs of the compound for Mycobacterium tuberculosis ranged from 0.00024 to 0.002 μg/ml. It had bactericidal activity against both growing and intracellular bacilli, and the frequency of spontaneous resistance for M. tuberculosis H37Rv was less than 1.

## TEXT

Tuberculosis (TB), an infectious disease caused by Mycobacterium tuberculosis, remains a leading public health threat (1). In 2017, there were an estimated 10.0 million new TB cases worldwide, and TB is currently the leading cause of death from infectious diseases, with 1.3 million and 300,000 TB deaths in HIV-negative and HIV-positive individuals, respectively (1). Furthermore, the emergence of multidrug-resistant (MDR) and extensively drug-resistant (XDR) TB, the dual epidemic of HIV-TB coinfection, the lack of adequate health care infrastructure, and the absence of an effective vaccine have all led to the continued persistence of the disease (2). Despite the fact that most TB cases are potentially curable, the prolonged nature of treatment regimens, particularly for drug-resistant TB, and the potential toxicity of many anti-TB agents further complicate the ability to eradicate the disease. Although three new agents, delamanid (DMD), pretomanid (PMD), and bedaquiline (BDQ) (3), have been approved for the treatment of MDR-TB in combinations with other TB drugs under varying conditions, additional new agents with novel mechanisms of action are still urgently needed to develop shorter, broadly active, and less toxic treatment regimens (4).

**FIG 1:**
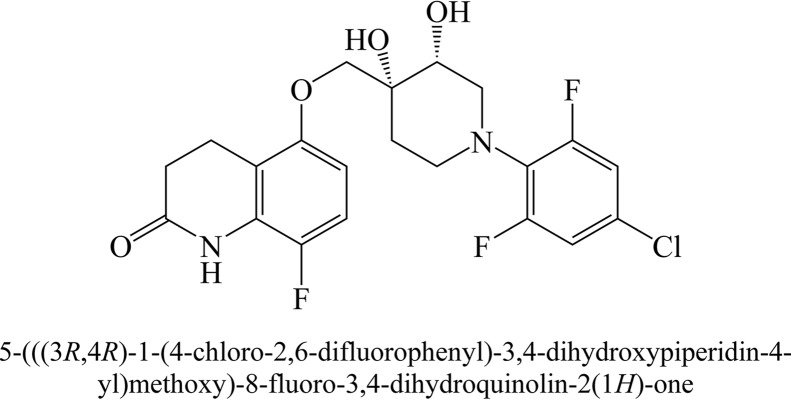
Structure and chemical name of OPC-167832.

As part of our ongoing TB drug discovery efforts, we used phenotypic screening methods to identify and optimize compounds with anti-TB activities from a core carbostyril structure, which has been recognized as having good absorption, distribution, metabolism, excretion, and toxicity profiles and has been used as the backbone of numerous drugs (5). These efforts led to a promising compound, OPC-167832 (Fig. 1), with potent anti-TB activities both *in vitro* and *in vivo*. Subsequently, we mapped the mode of action to the inhibition of decaprenylphosphoryl-β-d-ribose 2′-oxidase (DprE1), an essential enzyme for cell wall biosynthesis and a previously identified TB drug target (6). We report here the preclinical data for OPC-167832, including the *in vivo* efficacy of regimens composed of this compound and other new or repurposed anti-TB drugs.

## RESULTS

### *In vitro* antimycobacterial activity of OPC-167832.

As shown in Table 1, OPC-167832 exhibited very low MICs against laboratory strains of M. tuberculosis H37Rv and Kurono and strains with monoresistance to rifampin (RIF), isoniazid (INH), ethambutol (EMB), streptomycin (STR), and pyrazinamide (PZA). OPC-167832 has similar low MICs against other members of the M. tuberculosis complex (M. africanum, M. bovis, M. microti, M. caprae, and M. pinnipedii). These values are 10 to 1,000 times lower than those seen with other anti-TB agents, as shown in Table 1.

**TABLE 1:** MICs for M. tuberculosis complex standard strains^*a*^

Strain	MIC (μg/ml)						
	OPC-167832	DMD	RIF	BDQ	LVX	MXF	LZD
M. tuberculosis							
ATCC 27294 (H37Rv)	0.0005	0.004	0.25	0.063	0.5	0.5	1
ATCC 35812 (Kurono)	0.0005	0.004	0.25	0.063	0.5	0.13	1
ATCC 35838 (H37Rv RIF-R)	0.0005	0.004	>16	0.063	0.5	0.25	0.5
ATCC 35822 (H37Rv INH-R)	0.00024	0.004	0.5	0.063	0.5	0.25	1
ATCC 35837 (H37Rv EMB-R)	0.002	0.004	0.25	0.063	0.5	0.25	0.5
ATCC 35820 (H37Rv SM-R)	0.001	0.004	0.25	0.063	0.5	0.5	0.5
ATCC 35828 (H37Rv PZA-R)	0.001	0.004	1	0.13	0.5	0.5	1
M. africanum ATCC 25420	0.002	0.001	0.25	0.25	0.25	0.25	0.5
M. bovis ATCC BAA-935	0.002	0.004	0.063	0.13	0.25	0.13	0.5
M. caprae ATCC BAA-824	0.001	0.002	0.13	0.13	0.25	0.063	0.25
M. microti ATCC 11152	0.001	0.002	0.13	0.063	0.25	0.063	0.5
M. pinnipedii ATCC BAA-688	0.0005	0.002	0.5	0.13	0.25	0.25	1

a The MICs of OPC-167832 against M. tuberculosis complex standard strains were determined by an agar proportion method (CLSI document M24-A2 [35]). The MIC was determined as the lowest concentration of a compound inhibiting at least 99% of bacterial growth.

OPC-167832 also showed low MIC values against 40 clinically isolated M. tuberculosis strains, including 20 drug-susceptible, 14 MDR, and 6 XDR strains (see Table S2 in the supplemental material). OPC-167832 has minimal or no activity against standard strains of nonmycobacterial aerobic and anaerobic bacteria (data not shown), suggesting little effect on the resident bacterial flora of the host.

**FIG 2:**
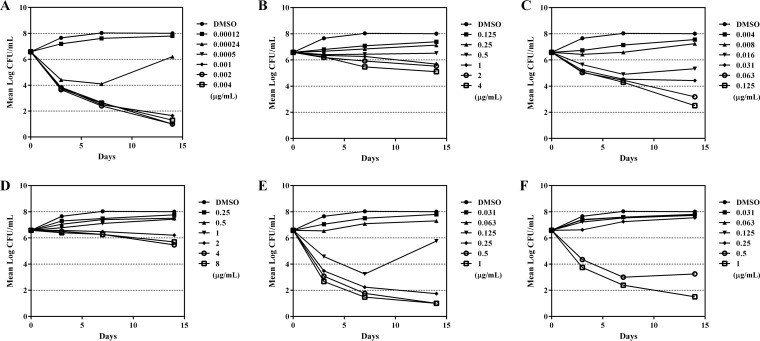
Time kill curves of OPC-167832 and other antituberculosis agents against growing bacilli. The bactericidal activities of OPC-167832 (A), BDQ (B), RIF (C), LZD (D), MXF (E), and LVX (F) at the indicated doses against growing bacilli were examined by counting CFU in culture media at 3, 7, and 14 days (*n* = 2).

Two experiments were conducted to compare the bactericidal activity of OPC-167832 with those of other anti-TB agents against growing or intracellular M. tuberculosis. As shown in Fig. 2, OPC-167832 exhibited bactericidal activity against growing M. tuberculosis H37Rv similar to that of RIF, moxifloxacin (MXF), or levofloxacin (LVX) but superior to that of BDQ or linezolid (LZD).

The killing activity of OPC-167832 was achieved at a very low concentration (0.0005 μg/ml), plateaued at high concentrations, and appeared to be time dependent. Similar results were obtained using M. tuberculosis strain Kurono (data not shown).

**FIG 3:**
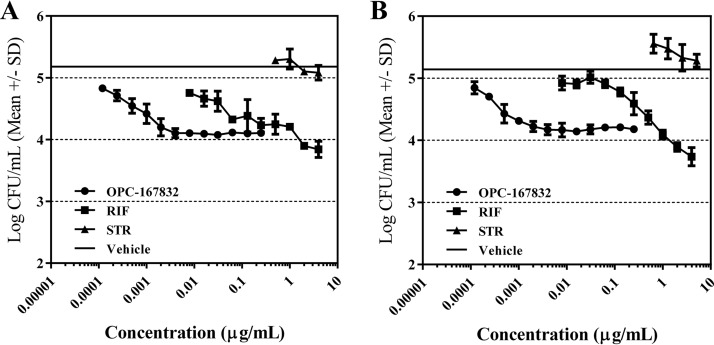
Bactericidal activity of OPC-167832 against intracellular bacilli. The bactericidal activity of OPC-167832 against M. tuberculosis H37Rv (A) and Kurono (B) intracellular bacilli was examined. Differentiated human THP-1 monocytic cells were inoculated with bacteria at a multiplicity of infection of 0.2 for 4 h. After removing the extracellular bacteria with 20 μg/ml of STR, the intracellular bacteria were treated with anti-TB agents at the indicated concentrations for another 3 days before bacteria were counted (*n* = 3). The solid horizontal lines indicate the CFU results for the vehicle group, which are independent of the drug concentration on the *x* axis. SD, standard deviation.

Against intracellular M. tuberculosis strains H37Rv and Kurono in THP-1-derived macrophage-like cells, OPC-167832 and RIF killed the intracellular bacilli, while STR exhibited no bactericidal activity (Fig. 3).

The 90% inhibitory concentration (IC_90_) values of OPC-167832 against intracellular M. tuberculosis strains H37Rv and Kurono were 0.0048 and 0.0027 μg/ml, respectively, which were lower than those of RIF (1.1501 and 0.5648 μg/ml, respectively). OPC-167832 showed bactericidal activity against intracellular M. tuberculosis at a low concentration, and the bactericidal activity was saturated at concentrations of 0.004 μg/ml or higher.

### Frequency of spontaneous resistance of M. tuberculosis to OPC-167832.

The frequencies of spontaneous resistance of five M. tuberculosis H37Rv clones to OPC-167832 at concentrations of 4-fold, 16-fold, 64-fold, or 256-fold of its MIC were 1.56 × 10^−8^ to 1.91 × 10^−7^, 2.60 × 10^−9^ to 1.52 × 10^−7^, 2.11 × 10^−9^ to 8.75 × 10^−8^, or <9.70 × 10^−10^ to <2.60 × 10^−9^, respectively. These values are comparable to or lower than those reported for DMD (4.2 × 10^−5^ to 6.4 × 10^−6^), BDQ (10^−8^ to 10^−7^), RIF (3.1 × 10^−8^), INH (3.5 × 10^−6^), LZD (10^−8^), and MXF (2.3 × 10^−8^) (7–11).

### Mode of action of OPC-167832.

Whole-genome DNA sequencing revealed mutations of two genes, *rv0678* and *rv3790*, in OPC-167832-resistant isolates (see Table S3 in the supplemental material). Rv0678 was reported to be a repressor for the MmpS5-MmpL5 efflux system of M. tuberculosis, and Rv3790 was reported as DprE1 (12–14).

By targeted sequencing, the mutations in the *rv0678* gene were confirmed in 4 weakly (4× MIC) resistant isolates and in the *rv3790* gene in 8 highly (16× to 256× MIC) resistant isolates (see Table S3). The loss of Rv0678 function causing low resistance to OPC-167832 was due to the insertion of nucleic acids into the *rv0678* gene, while the mutations in Rv3790 (DprE1) were found to be amino acid substitutions.

OPC-167832 inhibited the enzymatic activities of recombinant M. bovis BCG-derived DprE1 (100% identity with the amino acid sequences of M. tuberculosis DprE1), as shown in Table 2. The IC_50_ value of OPC-167832 was 0.258 μM versus 0.403 μM and 0.267 μM, respectively, for BTZ043 and PBTZ169, two known DprE1 inhibitors (13, 15, 16). INH, a non-DprE1 inhibitor, exhibited no activity against the recombinant DprE1 (IC_50_ > 10 μM).

**TABLE 2:** Inhibition of DprE1 enzymatic activity by OPC-167832, BTZ043, and PBTZ169^*a*^

Compound	IC_50_ (μM)		
	Mean	95% confidence interval	
		Lower	Upper
OPC-167832	0.258	0.222	0.295
BTZ043	0.403	0.329	0.489
PBTZ169	0.267	0.220	0.320
INH	>10		

a Enzymatic-inhibition assays were performed in duplicate on one 96-well plate, and the same assay was repeated on three independent plates. The IC_50_ was defined as the concentration at which 50% of DprE1 enzymatic activity was inhibited. The IC_50_ and corresponding 95% confidence intervals were determined using 4-parameter logistic regression analysis.

### Checkerboard assay of OPC-167832 in combination with other anti-TB agents.

**FIG 4:**
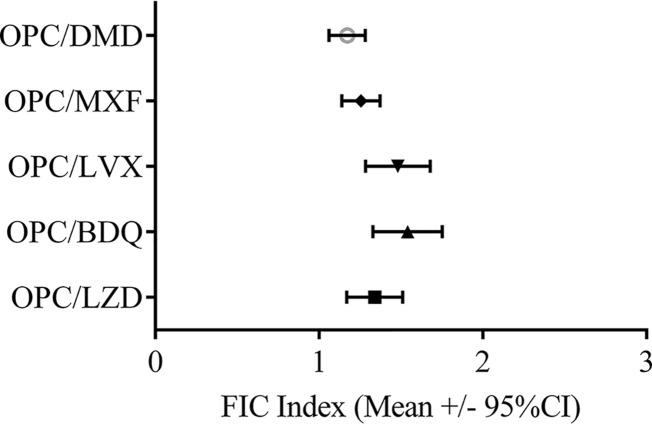
*In vitro* combination effects of OPC-167832 and other anti-TB agents (checkerboard assay). The *in vitro* combination effects of OPC-167832 and existing anti-TB agents against 27 strains of M. tuberculosis were examined by the checkerboard agar dilution method. CI, confidence interval.

We assessed the interactive effects of OPC-167832 in combination with other anti-TB agents by a checkerboard agar dilution method in order to assess the possibility of combining OPC-167832 with existing anti-TB agents. The mean fractional inhibitory concentration (FIC) index and the 95% confidence interval of each drug combination are shown in Fig. 4.

OPC-167832 showed an indifferent effect (i.e., no antagonism) when combined with DMD, MXF, LVX, BDQ, or LZD, indicating that OPC-167832 can be combined with the anti-TB agents tested *in vivo*.

### Pharmacokinetics of OPC-167832 in mice.

Before an *in vivo* study was conducted to evaluate the bactericidal activity of OPC-167832, we initially examined the pharmacokinetic (PK) profile of OPC-167832 at doses of 0.625, 1.25, 2.5, 5, and 10 mg/kg of body weight in healthy ICR female mice. After oral administration, the plasma OPC-167832 level reached peak at 0.5 h to 1.0 h (*t*_max_) and was eliminated with a half-life (*t*_1/2_) of 1.3 h to 2.1 h (see Table S4 in the supplemental material). OPC-167832 distribution in the lungs was approximately 2 times higher than that in plasma, and the *C*_max_ and AUC*_t_* (area under the concentration-time curve calculated to the last observable concentration at time *t*) of OPC-167832 in plasma and the lungs showed dose dependency (see Table S4).

### Therapeutic efficacy of OPC-167832 in experimental mouse models of chronic TB. (i) Efficacy of OPC-167832 monotherapy.

**FIG 5:**
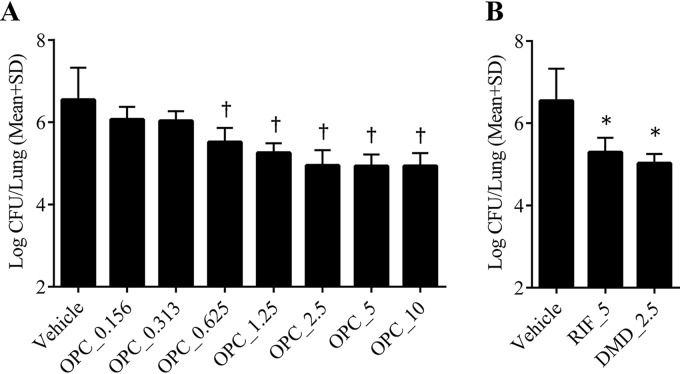
*In vivo* efficacy of OPC-167832 against a mouse chronic TB model. The viable bacteria in the lungs of M. tuberculosis Kurono-infected mice were counted after treatment with OPC-167832 (OPC; 0.156, 0.313, 0.625, 1.25, 2.5, 5, and 10 mg/kg) (A) or with RIF (5 mg/kg) plus DMD (2.5 mg/kg) (B) for 4 weeks at 7 days per week (*n* = 5). Each value represents the mean of results from five animals, and the error bars indicate SD from five replicates. Statistically significant differences between the RIF- or DMD-treated group and the vehicle control group were determined by Dunnett’s test (*, *P* < 0.01). The significance level of the test was set at 5% (two tailed), and the Williams test (2.5% lower tailed) was performed for confirmation of the dose dependency of OPC-167832 (†, *P* < 0.01).

As shown in Fig. 5A, OPC-167832 alone at doses of 0.625, 1.25, 2.5, 5, and 10 mg/kg significantly reduced lung CFU after 4 weeks of treatment compared to the vehicle group. The dose-dependent decrease of lung CFU was observed from 0.625 mg/kg to 2.5 mg/kg, and the bactericidal activity appeared to plateau at higher doses. The maximum efficacy of OPC-167832 was similar to those of reference agents at the indicated human-equivalent doses (RIF at 5 mg/kg and DMD at 2.5 mg/kg) (Fig. 5B).

### (ii) Efficacy of two-agent combination therapy.

**FIG 6:**
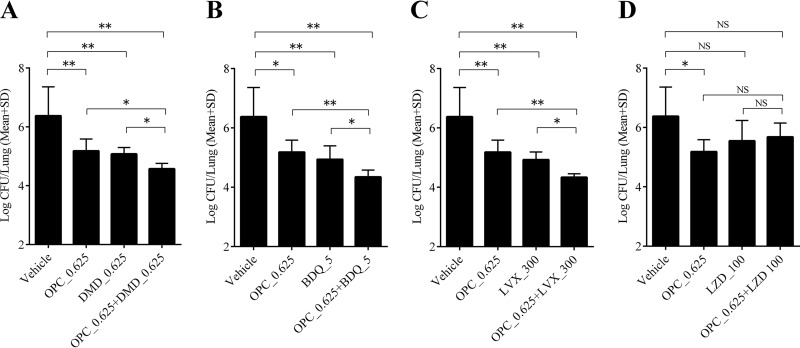
*In vivo* combination effect of OPC-167832 with DLM, BDQ, LVX, or LZD. The combination effect of OPC-167832 with DMD (A), BDQ (B), LVX (C), or LZD (D) in M. tuberculosis Kurono-infected ICR female mice was evaluated. Each value represents the mean of results from five samples, and the error bars are SD from five replicates. The significance level of the test was set at 5%. Statistically significant differences between the treated groups and the vehicle group were determined by Dunnett’s test. The significant differences between the combined treatment groups and their single-agent treatment groups were also determined by Dunnett’s test. *, *P* < 0.05; **, *P* < 0.01; NS, not significant.

As shown in Fig. 6, the activities of OPC-167832, DMD, BDQ, and LVX alone at the indicated doses were similar, and a significant decrease of lung CFU was observed in all the treatment groups compared to the vehicle group. LZD alone did not show a significant decrease. Two-agent combinations of OPC-167832 with DMD, BDQ, or LVX exhibited significantly higher efficacies than each single agent alone. On the other hand, no add-on effect was observed in the combination of OPC-167832 and LZD.

### (iii) Bactericidal and sterilizing activities of regimens containing delamanid and OPC-167832 as the core in a mouse model of chronic TB.

To investigate potential combination regimens containing DMD and OPC-167832 (DC) as a core component (i.e., DC-based regimens), we evaluated the therapeutic effects of DC as part of 3- or 4-drug combination regimens in TB-infected mice and compared them to that of the standard regimen, RHZE (RIF, INH, PZA, and EMB). We evaluated the following regimens by choosing LZD (Lz), MXF (M), or BDQ (B) as the third and/or fourth agent: DCLz, DCM, DCB, DCLzM, DCLzB, and DCMB. These new combination regimens (except for the DCLz regimen) were shown to produce more rapid decreases in lung CFU and higher proportions of mice that were culture negative in the lungs than the RHZE regimen (Table 3). The proportion of mice that were culture negative in the lungs at the end of treatment tended to be higher for the following three regimens that include DMD, OPC-167832, and MXF: DCM, DCLzM, and DCMB regimens. No obvious add-on effect of LZD in the DC, DCB, and DCM regimens was observed. Among these regimens, the DCMB regimen demonstrated the most potent efficacy: the lung CFU count after 6 weeks of treatment was below the detection limit, and at the end of just 8 weeks of treatment, the bacteria in the lungs of all the evaluated mice had already been eradicated (Table 3).

**TABLE 3:** Log_10_ CFU counts and proportions of mice with M. tuberculosis-negative cultures at the end of treatment

Group^*a*^	Log_10_ CFU/lung (no. culture negative/total no. of animals)^*b*^					
	Initial	4 wks	6 wks	8 wks	10 wks	12 wks
None	6.504 ± 0.155	NT	NT	NT	NT	NT
Vehicle	NT	6.454 ± 0.477	6.789 ± 0.329	6.761 ± 0.156 (0/5)	6.654 ± 0.706 (0/4)	6.491 ± 0.570 (0/5)
RHZE/RH	NT	3.846 ± 0.123	2.704 ± 0.330	1.389 ± 0.800 (0/5)	2.983 ± 0.697 (0/5)	0.314 ± 0.701 (1/5)
DC	NT	3.718 ± 0.412	3.083 ± 0.477	2.370 ± 0.183 (0/5)	1.819 ± 0.069 (0/5)	1.424 ± 0.195 (0/5)
DCLz	NT	3.957 ± 0.236	3.290 ± 0.140	2.258 ± 0.281 (0/5)	1.739 ± 0.202 (0/5)	1.455 ± 0.183 (0/5)
DCM	NT	2.520 ± 0.303	1.679 ± 0.442	0.298 ± 0.346 (0/5)	0.077 ± 0.130 (1/4)	0.000 ± 0.000 (5/5)
DCB	NT	2.393 ± 0.380	1.409 ± 0.135	0.295 ± 0.445 (1/5)	0.241 ± 0.538 (3/5)	0.468 ± 0.807 (3/5)
DCLzM	NT	3.221 ± 0.201	2.068 ± 0.382	0.328 ± 0.452 (1/4)	0.075 ± 0.151 (2/4)	0.012 ± 0.027 (4/4)
DCLzB	NT	2.648 ± 0.253	1.618 ± 0.456	0.354 ± 0.415 (1/3)	0.156 ± 0.348 (3/5)	0.241 ± 0.392 (3/5)
DCMB	NT	1.349 ± 0.004	1.352 ± 0.005	0.016 ± 0.022 (3/3)	0.000 ± 0.000 (5/5)	0.000 ± 0.000 (5/5)

a R, RIF (5 mg/kg); H, INH (25 mg/kg); Z, PZA (150 mg/kg); E, EMB (100 mg/kg); D, DMD (2.5 mg/kg); C, OPC-167832 (2.5 mg/kg); Lz, LZD (100 mg/kg); M, MXF (100 mg/kg); B, BDQ (25 mg/kg).

b ICR female mice were intratracheally infected with M. tuberculosis Kurono, and chemotherapy was initiated 14 days after infection for 4, 6, 8, 10, or 12 weeks at 5 days per week (*n* = 5, except for one contaminated sample each in the DCMB group at 4 weeks and the DCLzM group at 10 weeks and one dead mouse in each of the vehicle groups at 6 and 10 weeks due to mistakes in administration [*n* = 4]). NT, not tested. For the RHZE group, mice received a combination treatment of RHZE for the initial 8 weeks, followed by further treatment with RH for 4 weeks. Each value represents the mean ± standard deviation (SD), which was calculated using the individual-mouse data. For mice with one or more contaminated plates but with the rest of the plates containing no M. tuberculosis, we used a detection limit methodology, as described in Materials and Methods. For detailed calculations of mouse data using this methodology, see Table S5 in the supplemental material. The proportions of mice that were culture negative (defined as no bacteria detected in any of the plates with lung homogenates) in the lungs are presented in parentheses. There were contaminated plates with lung homogenates from several mice: one each in the DCM 10-week, DCLzM 8-week, and DCLzM 12-week groups and two each in the DCLzB 8-week and DCMB 8-week groups. M. tuberculosis was not detected in the rest of the plates from these mice, and we excluded them from the values in parentheses.

### (iv) Relapse-preventing effects of regimens containing delamanid and OPC-167832 as the core in a mouse model of chronic TB.

DCMB, identified as the most effective regimen in the bactericidal and sterilizing experiments, was examined in a relapse model. In this study, the proportions of mice with relapse at 12 weeks after the end of treatment with the DCMB regimen for 10, 12, and 14 weeks were compared to those for the standard regimen, RHZE, in the mouse model of chronic TB. As shown in Table 4, 2 weeks postinfection and prior to the initiation of treatment, the bacterial load in animal lungs reached 6.19 log_10_ CFU. Without drug treatment (vehicle control), lung bacterial loads stayed level. The DCMB regimen eliminated the bacteria in the lungs of mice with no relapse confirmed from 10 weeks of treatment onward. On the other hand, although the standard RHZE regimen also significantly reduced the number of TB bacilli in the lung over time, there were still 4 or 1 mice with viable bacteria after 12 or 14 weeks of treatment, respectively.

**TABLE 4:** Log_10_ CFU counts at the end of treatment and proportions of relapsed mice 12 weeks after treatment^*a*^

Regimen^*b*^	Log_10_ CFU/lung (mean ± SD; *n* = 5)						Proportion (%) of mice with relapse after treatment (*n* = 15)		
	Initial	4 wks	8 wks	10 wks	12 wks	14 wks	10 wks	12 wks	14 wks
None	6.190 ± 0.238	NT	NT	NT	NT	NT	NT	NT	NT
Vehicle	NT	6.348 ± 0.303	7.052 ± 0.595	6.667 ± 0.554	5.504 ± 0.193^*c*^	6.337 ± 0.629	NT	NT	NT
RHZE/RH^*d*^	NT	3.701 ± 0.243	0.947 ± 0.108^*c*^	0.685 ± 0.443	0.134 ± 0.084^*e*^	0.396 ± 0.445^*c*^	12 of 15 (80)	4 of 14 (29)^*c*^	1 of 14 (7)^*c*^
DCMB	NT	1.706 ± 0.414^*e*^	0.000 ± 0.000	0.038 ± 0.034^*e*^	0.086 ± 0.099^*e*^	0.044 ± 0.098^*e*^	0 of 15 (0)	0 of 14 (0)^*c*^	0 of 15 (0)

a ICR female mice were intratracheally infected with M. tuberculosis Kurono, and chemotherapy was initiated 14 days after infection for 4, 8, 10, 12, or 14 weeks at 5 days per week. Relapse was defined as a positive lung culture 12 weeks after the end of treatment for the indicated periods. NT, not tested.

b B, BDQ (25 mg/kg); C, OPC-167832 (2.5 mg/kg); D, DMD (2.5 mg/kg); E, EMB (100 mg/kg); H, INH (25 mg/kg); M, MXF (100 mg/kg); R, RIF (5 mg/kg); Z, PZA (150 mg/kg).

c One animal of each group died due to a mistake in administration.

d Z and E were administered only for the initial 8 weeks.

e Means and SD were calculated using the individual-mouse data. For mice with one or more contaminated plates but with the rest of the plates containing no M. tuberculosis, we used a detection limit methodology, as described in Materials and Methods. For detailed calculations of mouse data using this methodology, see Table S6 in the supplemental material.

## DISCUSSION

The development of shorter, less toxic, and more effective anti-TB regimens is not possible without new molecular entities against TB (3, 17). Recent approvals of DMD, PMD, and BDQ provide hope (3), but additional new agents are needed. Here, we present data to show that a new carbostyril derivative, OPC-167832, is a novel anti-TB drug candidate that can potentially be combined with DMD and other, newer anti-TB agents. OPC-167832 has the same carbostyril structure as several drugs in other therapeutic areas that are known to have good PK and safety profiles (5), thus providing hope that it may share the same favorable characteristics.

Our data show that OPC-167832’s anti-TB effect is likely the result of inhibition of DprE1, a known anti-TB drug target (15, 18–20). Mutations in DprE1 led to high-level resistance to OPC-167832 and other DprE1 inhibitors. There were four types of mutations in DprE1 reported here that produced moderate (Gly248Cys, Asn364Ser, and Cys387Ser) to high-level (Tyr314His) resistance to OPC-167832 (see Table S4). Interestingly, only the substitution of cytosine for thymine at nucleotide position 940 (which leads to Tyr314His) was found in all three isolates exhibiting high-level resistance (256 times the MIC versus the wild type). Of note, Tyr-314 is located at an active pocket of DprE1, and a mutation of this amino acid has been related to resistance to TCA1, a DprE1 inhibitor currently in development (19). OPC-167832 has anti-TB activity at very low concentrations comparable to those of BTZ043 and its derivatives against growing M. tuberculosis. While a mutation at Cys387 on DprE1 leads to high-level BTZ043 resistance (1,560 to >12,500 times the MIC versus the wild type), a mutation in the same position produces only moderate resistance to OPC-167832 (16 times the MIC versus the wild type) (see Table S3), suggesting distinct means of DprE1 binding (i.e., covalent versus noncovalent binding) (18, 21, 22) may affect the level of resistance.

Our results indicate that M. tuberculosis has at least two resistance mechanisms against OPC-167832, with different degrees of resistance, probably a non-target-based mechanism (mutations in the *rv0678* gene) and a target-based mechanism (mutations in the *rv3790* gene). The involvement of Rv0678 in developing OPC-167832 resistance raises the possibility of cross-resistance with other drugs, such as BDQ and clofazimine (23). Although the MIC of OPC-167832 against Rv0678 mutants was still low (0.001 μg/ml) and the exposure after an oral dose was much higher, as in the mouse study (314-fold for *C*_max_/MIC and 95-fold for AUC*_t_*/12 h/MIC at a dose of 2.5 mg/kg [see Table S4]) and preliminary human studies (unpublished data), we plan to further study the killing by OPC-167832 of this mutant in an animal model. Importantly, the development of efflux pump-related low-level resistance and resultant possible cross-resistance to other TB drugs need to be carefully evaluated in future clinical studies.

The minimum effective dose of OPC-167832 was very low, and the efficacy of 2.5 mg/kg OPC-167832 was almost equivalent to those of DMD and RIF at clinically relevant doses (Fig. 5). OPC-167832 showed therapeutic effect at lower doses (0.625 to 10 mg/kg) than other DprE1 inhibitors, such as TCA1 and PBTZ169, in mice. TCA1 has an MIC of 0.19 μg/ml (19) and thus is a much weaker DprE1 inhibitor. While PBTZ169 and OPC-167832 have similar MIC ranges, PBTZ169 tends to have a much lower distribution in the lungs of mice (*K_p_* [tissue to plasma partition coefficient] = 0.415) (24) than OPC-167832 (*K_p_* range, 1.67 to 1.96 [see Table S4]). These may be the reasons that high doses of both TCA1 and PBTZ169 are necessary to obtain the exposure required to demonstrate sufficient efficacy (15, 19). In combination studies using DMD and OPC-167832 as the core, we selected three agents, BDQ, MXF, and LZD, as candidates to construct regimens, as these three agents have higher potencies than the other drugs used to treat MDR-TB (25). In general, the efficacies of the DC-based regimens were similar and/or superior to that of the RHZE regimen. However, the add-on effect of LZD was not observed when comparing the efficacies of DCLz and DCLzM regimens to those of DC and DCM regimens. The reasons for the lack of an LZD add-on effect are unclear, but a similar phenomenon was observed in the two-drug combination therapy study in mice. Also, a previous study by Williams et al. (26) reported that the combination of LZD and RHZ (RIF, INH, and PZA) was less effective than an RHZ regimen in a mouse model of chronic TB and that LZD, when administered simultaneously with RHZ, affected the PK profiles of the combined agents in the same animals. The PK profiles of our compounds with LZD need further examination.

Of note, the DCMB regimen was the most effective among the tested DC-based regimens. The lung bacterial counts after treatment with the DCMB regimen decreased most rapidly, and conversion of the lung bacterial counts of all the mice to negative status was achieved after 8 weeks of treatment. Most important, the DCMB regimen-treated mice had no relapse after 10 weeks of treatment.

Our findings highlight the potential anti-TB activity of OPC-167832 *in vitro* and *in vivo*. However, there are limitations to the present study. When we evaluated the combination effects of OPC-167832 with DMD or BDQ, we used only doses lower than the human-equivalent ones due to the potent efficacies of the two drugs alone in the mouse model. Because of the use of lower exposures than in humans, the effect of OPC-167832 combined with these drugs in clinical use may not be fully reflected. Moreover, we have not evaluated the contributions of individual drugs in the three- or four-drug combination regimens specifically, and only a limited number of drugs were combined. Ongoing and future studies will determine the contribution of each drug and, ideally, the optimal dose for each drug in a combination. Obtaining such information will likely require a combination of nonclinical and clinical studies. Lastly, the relapse rate was studied in only one mouse model, for which the translational value to patients is still being debated (27–29). While mice are the most commonly used species for efficacy evaluation in TB drug development and data obtained from mice have contributed to an understanding of the roles of current TB drugs, the main challenge is that human pulmonary TB disease is very heterogeneous, and traditional mouse strains, such as the one used in this study, do not well mimic the TB pathological characteristics observed in humans, such as the lack of caseous necrosis in the traditional mouse models (30). A newer mouse model using the C3HeB/FeJ strain has been shown to develop TB lesions closer to human pathologies (30); however, the model has large variations (31, 32) and has not been used widely in relapse studies. An additional challenge is the shift of TB drug development from developing a single drug to developing a regimen to shorten the treatment duration (28). However, the current preclinical models do not have a strong track record in predicting treatment duration in humans, and there is no consensus on which models should be used (27, 28). As we learn more about preclinical models and their predictive accuracy in regard to regimen development, additional studies should be conducted to further evaluate the OPC-167832-containing regimens.

These limitations notwithstanding, our study suggests that the newly synthesized carbostyril derivative OPC-167832 has a mode of action for inhibiting DprE1 enzymatic activity against M. tuberculosis that is different from those of currently marketed anti-TB drugs. The combination of OPC-167832 with other new or repurposed anti-TB drugs, especially the regimen comprised of OPC-167832, DMD, BDQ, and MXF, exhibited excellent outcomes in relapse prevention in a mouse TB model. Therefore, OPC-167832 appears to be a promising component in the development of new regimens for TB treatment. To this end, OPC-167832 has entered clinical development, and a phase 1b/2a trial to evaluate its safety and efficacy in pulmonary TB subjects is ongoing (33).

## MATERIALS AND METHODS

### Antibiotics.

INH, RIF, PZA, EMB, and STR were purchased from Sigma-Aldrich. LVX, MXF, LZD, and BDQ were purchased from Tokyo Chemical Industry Co., Ltd.; Kemprotec, Ltd.; Pfizer Japan, Inc.; and NARD Institute, Ltd., respectively. DMD, OPC-167832, BTZ043, and PBTZ169 were synthesized in house by Otsuka Pharmaceutical Co., Ltd. All antibiotics were dissolved and diluted with dimethyl sulfoxide (DMSO) or distilled water in *in vitro* assays and were suspended or dissolved in a 5% (wt/vol) gum arabic solution in *in vivo* experiments.

### Bacterial strains and cell line.

M. tuberculosis strains ATCC 27294 (H37Rv), ATCC 35812 (Kurono), ATCC 35838 (H37Rv RIF-R), ATCC 35822 (H37Rv INH-R), ATCC 35837 (H37Rv EMB-R), ATCC 35820 (H37Rv SM-R), and ATCC 35828 (H37Rv PZA-R); M. africanum ATCC 25420; M. bovis ATCC BAA-935; M. caprae ATCC BAA-824; M. microti ATCC 11152; and M. pinnipedii ATCC BAA-688 were purchased from the American Type Culture Collection. M. bovis BCG Tokyo was obtained from the Laboratory of Culture Collection, Institute of Medical Science, University of Tokyo. A total of 40 M. tuberculosis clinical strains, including 20 MDR/XDR strains (see Table S1 in the supplemental material), used in this study were isolated in Japan, Myanmar, Thailand, Cambodia, Indonesia, Mongolia, Philippines, Yemen, Korea, Estonia, Latvia, Lithuania, Vietnam, and China. The human monocytic leukemia THP-1 cell line was purchased from Dainippon Pharmaceutical Co., Ltd.

### Culture media and conditions.

All the bacterial strains were grown at 37°C in Middlebrook 7H9 broth (Becton Dickinson) containing 10% albumin-dextrose-catalase (ADC) enrichment (Becton Dickinson) and 0.05% Tween 80 or on Middlebrook 7H11 agar medium (Becton Dickinson) containing 10% oleic acid-albumin-dextrose-catalase (OADC) enrichment (Becton, Dickinson) and 0.5% glycerol. Antibiotics were added to the indicated concentrations if needed. For the CFU count in *in vivo* studies, 0.4% (wt/vol) activated charcoal was added to Middlebrook 7H11 agar medium to minimize the carryover of anti-TB agents (34). THP-1 cells were grown in RPMI 1640 medium (Sigma-Aldrich) containing 10% fetal bovine serum at 37°C with 5% CO_2_.

### Susceptibility testing.

Either an agar proportion method (35) or an agar dilution method (36) was used to measure the MICs of anti-TB agents against standard strains of the M. tuberculosis complex and clinical strains of M. tuberculosis.

### Checkerboard assay.

The checkerboard procedure was performed using the MICs of tested agents against the bacterial strains established by an agar dilution method reported previously (36). The level of synergy was determined by calculating the FIC index based on the following formulas: ΣFIC = FIC of compound A + FIC of compound B; FIC of compound A = MIC of compound A in combination ÷ MIC of compound A alone; FIC of compound B = MIC of compound B in combination ÷ MIC of compound B alone. The average ΣFIC value was interpreted using the European Committee on Antimicrobial Susceptibility Testing criteria (37) with the following values: ≤0.5, synergy; >0.5 to 1, additive effect; >1 to 2, indifference; >2, antagonism). The averages of the ΣFIC values and the 95% confidence intervals for the tested bacterial strains were calculated.

### Bactericidal activity.

To evaluate the bactericidal activity against growing bacteria, anti-TB agents were added to 5 ml of M. tuberculosis H37Rv and Kurono cultures to reach the final concentrations shown in Fig. 2. The bacteria were incubated at 37°C for 3, 7, or 14 days and then harvested for viable-bacteria counts on Middlebrook 7H11 agar plates.

The bactericidal activity of OPC-167832 against M. tuberculosis strains H37Rv and Kurono in THP-1 cells differentiated by 0.1 μg/ml of phorbol 12-myristate 13-acetate for 48 h was determined by a method described previously (36).

### Frequency of spontaneous resistance to OPC-167832.

Single colonies isolated from M. tuberculosis H37Rv were cultured in Middlebrook 7H9 broth, and the bacterial suspensions (10^8^ to 10^9^ total CFU) were cultured on Middlebrook 7H11 agar plates containing DMSO or OPC-167832 at concentrations of 4, 16, 64, and 256 times the MIC to determine the numbers of resistant and viable bacteria in the suspension. The frequency of spontaneous resistance was calculated for each group as the number of resistant colonies divided by the number of inoculated bacteria.

### Determination of mutant genes from resistant isolates of M. tuberculosis.

The genomic DNAs of a sensitive isolate of M. tuberculosis H37Rv and six OPC-167832-resistant isolates from the experiment to determine the frequency of spontaneous resistance to M. tuberculosis H37Rv were extracted by a standard protocol (38). Sequencing of isolates was performed using HiSeq2500 from Illumina (100 bp; paired end). The HiSeq run yielded an average quality score of over Q38, with >96% of the bases being more than Q30. The paired-end raw reads of each isolate were mapped to the parental M. tuberculosis H37Rv reference genome (GenBank accession no. NC_000962) using Burrows-Wheeler Aligner version 0.7.10. Single nucleotide polymorphisms (SNPs) on mutant genes were called using Genome Analysis Toolkit (GATK) version 2.3.0 and were confirmed by Sanger sequencing using the primers listed in Table S1. Furthermore, according to the SNP analysis results, the sequences of two genes of 12 OPC-167832-resistant isolates and 1 OPC-167832-sensitive isolate were also confirmed by Sanger sequencing.

### Preparation of recombinant DprE1.

M. bovis BCG Tokyo *dprE1* was cloned into pET SUMO (Invitrogen) according to the method in a previous report (39). Recombinant DprE1 fused to an N-terminal His6-SUMO tag was coexpressed with chaperones from Escherichia coli (GroES) in E. coli strain BL21(DE3) for 24 h h at 16°C (after induction with 0.5 mM isopropyl beta-d-thiogalactoside). After the cells were disrupted, the lysate was loaded onto a 1-ml HisTrap, and the protein was eluted with 250 mM imidazole. Tag cleavage was achieved by overnight incubation with SUMO protease, followed by a second HisTrap purification to remove both the tag and SUMO protease. After a buffer exchange, the protein solution was loaded on a 1-ml HiTrap Q HP column. The recombinant DprE1 was eluted with a gradient of 20 mM NaCl steps from 100 mM to 200 mM in buffer B and concentrated using an Amicon ultra filter (10-kDa cutoff).

### DprE1 inhibition assay.

The inhibition assay for DprE1 was performed following the method of Makarov et al. (40), using a coupled Amplex Red-horseradish peroxidase assay. Farnesyl-phosphoryl-β-d-ribofuranose, which is a decaprenylphosphoryl-β-d-ribose (DPR) analog, was used as the substrate instead of DPR. BTZ043 and PBTZ169, two reported DprE1 inhibitors (15, 18), were used as positive controls, and their activities were compared with that of OPC-167832. INH, an InhA inhibitor (41), was used as a non-DprE1 inhibitor control. Enzymatic-inhibition assays were performed in duplicate on one 96-well plate, and the same assay was repeated using three independent plates.

### Experimental animals.

This study was carried out in adherence to the Guidelines for Animal Care and Use (42), which were approved by the Animal Care and Use Committee of Otsuka Pharmaceutical Co., Ltd.

Five-week-old female mice (Slc:ICR) with body weights ranging from 22 to 24 g (purchased from Japan SLC, Inc.) were housed in Clean S-PSF cages (Clea Japan, Inc.) and given at least 3 days to acclimate to the housing facility. The mice were specific-pathogen-free grade and were housed with free access to a certified diet (MF; Oriental Yeast Co., Ltd) and water in our animal laboratories, in which environmental controls were set to the following conditions: temperature , 23°C ± 2°C; humidity, 60% ± 10%; and a 12-h light-dark cycle (light period, 7 a.m. to 7 p.m.). The mice were randomized to each group after determination of body weight in each experiment.

### Pharmacokinetics of OPC-167832 in mice.

Three uninfected mice from each group were given a single oral dose of 0.625, 1.25, 2.5, 5, or 10 mg/kg OPC-167832. The mice were anesthetized under isoflurane for blood sample collection at 0.5 h, 1 h, 2 h, 4 h, 6 h, 8 h, 12 h, or 24 h after dosing and then euthanized by exsanguination, and the lungs were collected. Heparinized blood samples were centrifuged at 880 × *g* for 10 min at 4°C to obtain the plasma. The lung samples were rinsed gently with ice-cold physiological saline, weighed, and immediately homogenized with a Shake Master Neo (Bio Medical Science Inc.) with two tungsten beads after adding a 9-fold volume of methanol-physiological saline (3:1 [vol/vol]). All plasma and lung samples were frozen and stored below −60°C until analysis. The samples were analyzed with a validated TSQ Quantum Ultra liquid chromatography-tandem mass spectrometry (LC–MS-MS) system (Thermo Fisher Scientific KK) for plasma and a validated 4000 QTrap LC–MS-MS system (AB Sciex) for lung homogenate. We determined the following PK parameters with WinNonlin Professional software (version 6.3; Pharsight Corp.): *C*_max_, peak (maximum) concentration of OPC-167832; *t*_max_, time to peak (maximum) concentration; AUC*_t_*, area under the concentration-time curve calculated to the last observable concentration at time *t*; and *t*_1/2_, terminal-phase elimination half-life.

### Therapeutic efficacy.

To evaluate the therapeutic efficacy of OPC-167832 and other anti-TB agents, we designed four mouse experiments, as described below. Mice were anesthetized by an intramuscular administration of 0.05 ml of ketamine-xylazine solution (Ketalar [Daiichi Sankyo Co., Ltd.], Selactar 2% [Bayer], and sterile physiological saline solution [8:3:9]) (43) and infected by intratracheal inoculation of a cryopreserved bacterial suspension of M. tuberculosis Kurono. The infected mice were housed for 4 weeks (except for the regimen experiment, which was for 2 weeks) and randomly assigned to groups prior to the initiation of treatment. At treatment initiation, one group of mice were sacrificed for baseline lung CFU count determination. Test agents for oral gavage were prepared weekly and stored separately in a 4°C refrigerator. For combination studies, on the day of administration, individually prepared agents were mixed and administered at once. The volume of each drug administration was calculated to be 10 ml/kg of average body weight (measured once a week). At designated time points, animals were sacrificed under anesthesia with isoflurane, and the lungs were aseptically excised, weighed, and homogenized with a Multi-beads shocker (Yasui Kikai Co.) with 5 ml of sterilized distilled water in the monotherapy and 2-agent combination studies or 2 ml of sterilized distilled water in the regimen studies. Lung homogenates or their 10-fold serial dilutions (with sterilized distilled water) were plated on 7 or 10 Middlebrook 7H11 agar plates containing 0.4% activated charcoal. The plates were incubated for 2 to 4 weeks at 37°C before the colonies were counted.

To calculate the number of CFU per lung for each mouse, CFU were summed from each plate. If contamination that prevented colony counting was detected in one or more of the plates while the rest contained countable colonies, the contaminated plate was excluded from the calculation. In this situation, CFU were summed from the countable plates and then divided by the total volume spread on the countable plates to obtain the number of CFU per milliliter, which was then multiplied by the total initial plated volume and the dilution factor to obtain the number of CFU per lung for each mouse. For mice treated for 8 weeks or longer, due to the expected low CFU counts, we plated the whole-lung homogenates without dilutions. In these mice, if all plates had no CFU, they were considered culture negative (CFU = 0). However, if contamination was detected in one or more of the plates while the rest of the plates had no M. tuberculosis colonies, one colony was assigned as the total CFU count of uncontaminated plates. The number of CFU per lung was then calculated as follows: 1 CFU divided by the total volume of lung homogenates plated on the uncontaminated plates, which was then multiplied by the total volume of the original lung homogenates. This value was considered the detection limit of our CFU-counting methodology. A detailed explanation of the calculation of the number of CFU per lung in groups containing such mice is provided in Tables S5 and S6 in the supplemental material. The slight difference in the lung weight of each mouse resulted in a slight difference in the detection limit. For data presentation, the number of CFU per lung obtained for each mouse was log transformed. In the calculation of culture negativity (number of animals culture negative divided by the total number of animals) (Table 3) and proportion (percentage) of mice with relapse after treatment (Table 4), we excluded mice with any number of contaminated plates.

### Monotherapy in a mouse model of chronic TB.

Mice received one of the following oral treatments daily for 4 weeks (*n* = 5): a vehicle control; DMD at 2.5 mg/kg; RIF at 5 mg/kg; or OPC-167832 at 0.156, 0.313, 0.625, 1.25, 2.5, 5, or 10 mg/kg. Doses for DMD and RIF were chosen to achieve human-equivalent levels in plasma (36).

### Two-agent combination therapy in a mouse model of chronic TB.

Mice (*n* = 5 in each treatment group) received one of the following oral treatments 5 days per week for 4 weeks: a vehicle control, OPC-167832 at 0.625 mg/kg, DMD at 0.625 mg/kg, a DMD (0.625 mg/kg)–OPC-167832 (0.625 mg/kg) combination, BDQ at 5 mg/kg, a BDQ (5 mg/kg)–OPC-167832 (0.625 mg/kg) combination, LVX at 300 mg/kg, an LVX (300 mg/kg)–OPC-167832 (0.625 mg/kg) combination, LZD at 100 mg/kg, or an LZD (100 mg/kg)–OPC-167832 (0.625 mg/kg) combination. Lower-than-human-equivalent doses were chosen for DMD and BDQ (36, 44) to allow room to detect possible additive or synergistic effects when the two drugs were combined, due to the potent efficacy already shown by the compounds alone. The minimum effective dose of OPC-167832, 0.625 mg/kg, was chosen, while the human-equivalent doses for LVX and LZD (44, 45) were used in the experiment.

### Bactericidal and sterilizing activities of regimens containing delamanid and OPC-167832 as the core in a mouse model of chronic TB.

The dosing frequency was 5 days per week for 4, 6, 8, 10, or 12 weeks. Mice (*n* = 5 in each treatment group) received one of the following oral treatments: a vehicle control, a standard TB regimen of RHZE (RIF, INH, PZA, and EMB), a DC regimen (DMD and OPC-167832), a DCLz regimen (DMD, OPC-167832, and LZD), a DCM regimen (DMD, OPC-167832, and MXF), a DCB regimen (DMD, OPC-167832, and BDQ), a DCLzM regimen (DMD, OPC-167832, LZD, and MXF), a DCMB regimen (DMD, OPC-167832, MXF, and BDQ), or a DCLzB regimen (DMD, OPC-167832, LZD, and BDQ). The mixtures for oral administration were prepared daily. The following doses were used: 2.5 mg/kg for OPC-167832, 2.5 mg/kg for DMD, 25 mg/kg for BDQ, 100 mg/kg for LZD, 100 mg/kg for MXF, 5 mg/kg for RIF, 25 mg/kg for INH, 100 mg/kg for EMB, and 150 mg/kg for PZA. The doses of these anti-TB agents were based on the suggested human-equivalent doses (36, 44), except for OPC-167832. For the RHZE group, mice received combination treatment of RHZE for the initial 8 weeks, followed by further treatment with RH for 4 weeks.

### Relapse-preventing effect of regimens containing delamanid and OPC-167832 as the core in a mouse model of chronic TB.

The dosing frequency was 5 days per week for 8, 10, 12, or 14 weeks. The mice received one of the following oral treatments (*n* = 5 for the groups used to evaluate bacterial numbers in the lungs just after treatment; *n* = 15 for the groups used to evaluate the relapse rate): a vehicle control, a standard TB regimen of RHZE (RIF, INH, PZA, and EMB), or a DCMB regimen (DMD, OPC-167832, MXF, and BDQ). For the RHZE group, mice received combination treatment with RHZE for the initial 8 weeks, followed by further treatment with RH. The dose and the preparation method for each agent and the method for efficacy evaluation were the same as those in the above-mentioned regimen therapy study. In this study, the proportions of mice that relapsed at 12 weeks after the end of treatment with the DCMB regimen for 10, 12, and 14 weeks were compared to those for the standard regimen, RHZE, in the mouse model of chronic TB.

### Statistical analysis.

Statistical analyses were conducted using SAS software (SAS Institute Japan; release 9.3). The significance level of the test was set at 5%.

IC_50_ and IC_90_ values and 95% confidence intervals for *in vitro* studies were determined by 4-parameter logistic or linear regression analysis, respectively. The sample size used in the *in vivo* studies allowed the detection of a 2-log-unit reduction difference, with a power of 0.80 and an alpha level of 0.05, and the Williams test (2.5% lower tailed) was performed to confirm the dose dependency of OPC-167832. The number of CFU per lung for each mouse was log transformed, and the statistically significant difference between the treated and vehicle control groups, or the combined treatment groups and their single-agent treatment groups, was determined by Dunnett’s test alone. For the regimen experiment, an average log CFU per lung value and the standard deviation for each regimen group were calculated.

## Supplementary Material

Supplemental file 1
