# Supplementary material for: OPC-167832, a Novel Carbostyril Derivative with Potent Antituberculosis Activity as a DprE1 Inhibitor
Source: Antimicrob Agents Chemother. 2020 May 21;64(6):e02020-19. doi: 10.1128/AAC.02020-19 (PMC7269503; doi:10.1128/AAC.02020-19)
Supplement: Supplemental file 1 [file AAC.02020-19-s0001.pdf]

Supplemental Material

TABLE S1 Primers Used in This Study

Primers	Sequence
For cloning of rv0678 and dprE1	
rv0678-F1	5’- TTATCGATGTGAGCGTCAACGACGGGGT -3’
rv0678-R1	5’- CCGGATCCTCAGTCGTCCTCTCCGGTTC -3’
dprE1-F	5’- ATGTTGAGCGTGGGAGCTACCACTA-3’
dprE1-R	5'- ATGTTGAGCGTGGGAGCTACCACTA-3'
For sequencing of dprE1	
dprE1-F2	5’- CAGCATGGACCTGCTGACCG -3’
dprE1-F3	5’- AACGGGCTGGCCAACAAATA -3’
dprE1-R2	5’- TATTTGTTGGCCAGCCCGTT -3’

TABLE S2 Antimycobacterial Activity of OPC-167832 Against Susceptible and MDR/XDR Strains

Group Name	No. of Strains	MIC (μg/mL)		
		Range	MIC_{50}	MIC_{90}
Susceptible	20	0.00024 - 0.002	0.0005	0.001
MDR/XDR	20	0.00024 - 0.002	0.001	0.002

The MICs of OPC-167832 against 40 strains of clinically isolated MTB, 20 strains of drug-susceptible and 20 strains of drug-resistant (14 MDR-TB and 6 XDR-TB), were determined by an agar proportion method (CLSI document M24-A2). The MIC to inhibit the growth of 50% and 90% of the isolates in each group (MIC_{50} and MIC_{90}) were also determined.

TABLE S3 Mutations on *rv0678* and *rv3790* Genes of OPC-167832-Resistant Strains

Isolation			MIC (µg/mL)	rv0678 (Transcriptional regulator)	rv3790 (DprE1)
MTB H37Rv	DMSO	-	0.00024	WT*	WT
	OPC-167832 4 x MIC	1	0.001	84_85insIS6110**	WT
		2	0.001	198_199insG (frameshift)	WT
		3	0.001	84_85insIS6110**	WT
		4	0.001	84_85insIS6110**	WT
	OPC-167832 16 x MIC	1	0.008	WT	742G→T (Gly248Cys)
		2	0.008	WT	1091A→G (Asn364Ser)
		3	0.004	WT	1160G→C (Cys387Ser)
		4	0.008	WT	1091A→G (Asn364Ser)
		5	0.004	WT	1091A→G (Asn364Ser)
	OPC-167832 64 x MIC	1	0.063	WT	940T→C (Tyr314His)
		2	0.063	WT	940T→C (Tyr314His)
		3	0.063	WT	940T→C (Tyr314His)

*: Corresponding to H37Rv wild type sequence

**: An insertion mutant which an IS6110 IS-like element of MTB H37Rv was inserted (1).

TABLE S4 Plasma and Lung Pharmacokinetic Parameters of OPC-167832 in Mice

Dose (mg/kg)	Plasma				Lung			
	Cmax (ng/mL)	tmax (h)	AUCt (ng*h/mL)	$t_{1/2}$[a] (h)	Cmax (ng/g)	tmax (h)	AUCt (ng*h/g)	$t_{1/2}$[a] (h)
0.625	912	1.0	283 (t: 8h)	1.6 (2 - 8h)	162	1.0	505 (t: 8h)	1.4 (2 - 8h)
1.25	200	1.0	543 (t: 12h)	1.5 (2 - 12h)	303	1.0	909 (t: 8h)	2.0 (4 - 8h)
2.5	314	0.5	1141 (t: 12h)	1.3 (6 - 12h)	651	0.5	2133 (t: 8h)	1.3 (2 - 8h)
5	758	1.0	2649 (t: 12h)	2.1 (2 - 12h)	1364	1.0	4742 (t: 12h)	2.1 (2 - 12h)
10	1094	1.0	3785 (t: 12h)	1.6 (2 - 12h)	2223	1.0	7410 (t: 12h)	2.1 (6 - 12h)

[a]$t_{1/2}$ in plasma and lung were calculated using the data at the time points indicated in parentheses.

TABLE S5 Calculation of Log_{10} CFU/lung in the study of bactericidal and sterilizing activities of regimens containing delamanid and OPC-167832.

This table contains data from individual mouse treated 8 weeks or longer and had contaminated plates. For these mice, lung homogenates were plated on 7 or 10 agar plates. But since one or more plates were contaminated, it prevented a direct summation of the total CFU counts. If contamination that prevented colony counting was detected in one or more of the plates while the rest contained countable colonies, the contaminated plate was excluded from the calculation. In this situation, CFUs were summed from countable plates, then divided by the total volume spread on these countable plates to obtain CFU/mL, which was then timed with the total initial plated volume and the dilution factor to obtain the CFU/lung for each mouse. In these mice, if all plates had no CFU, they were considered culture negative (CFU = 0). However, if contamination was detected in one or more of the plates while the rest of the plates had no MTB colonies, one colony was assigned as the total CFU count of uncontaminated plates. The CFU/lung was then calculated as follows: 1 CFU divided by the total volume of lung homogenates plated on the uncontaminated plates, which was then timed with the total volume of the original lung homogenates. This value was considered as the detection limit of our CFU counting methodology, as described in the Method section. Values obtained using this detection limit methodology have a prefix of <. The mean and SD data obtained in this table are shown in Table 3.

Animal ID	(A) Total volume of lung homogenate (mL)	Number of 7H11 plates	Number of contaminated plates	(B) Sum of lung homogenate spread on uncontaminated plates (mL)	(C) Number of detected colony	CFU/mL (C)÷(B)	CFU/lung (C)÷(B)×(A)	Log_{10} CFU/lung
Group: DCM-10w Mean: 0.077, SD 0.130								
1	2.18	7	1	1.80	0	<0.556[a]	<1.211[a]	<0.083[a]
2	2.22	7	0	2.22	0	0.000	0.000	0.000
3	2.18	7	0	2.18	1	0.4587	1.000	0.000
4	2.20	7	0	2.20	1	0.4545	1.000	0.000
5	2.20	7	0	2.20	2	0.9091	2.000	0.301

Group: DCLzM-8w Mean 0.328, SD 0.452								
1	2.21	10	0	2.21	0	1.810	4.000	0.602
2	2.21	10	1	2.01	9	4.478	9.896	0.995
3	2.25	10	0	2.25	1	0.4444	1.000	0.000
4	2.21	10	0	2.21	0	0.000	0.000	0.000
5	2.21	10	1	2.01	0	<0.498[a]	<1.100[a]	<0.041[a]
Group: DCLzM-12w Mean 0.012, SD 0.027								
1	2.25	7	0	2.25	0	0.000	0.000	0.000
2	2.22	7	0	2.22	0	0.000	0.000	0.000
3	2.20	7	0	2.20	0	0.000	0.000	0.000
4	2.26	7	0	2.26	0	0.000	0.000	0.000
5	2.28	7	1	1.98	0	<0.505[a]	<1.152[a]	<0.061[a]
Group: DCLzB-8w Mean 0.354, SD 0.415								
1	2.24	10	1	2.04	3	1.471	3.294	0.518
2	2.22	10	1	2.02	0	<0.495[a]	<1.099[a]	<0.041[a]
3	2.27	10	3	1.40	0	<0.714[a]	<1.621[a]	<0.210[a]
4	2.25	10	0	2.25	0	0.000	0.000	0.000
5	2.20	10	0	2.20	10	4.545	10.00	1.000
Group: DCMB-8w Mean 0.016, SD 0.022								
1	2.26	10	1	2.06	0	<0.485[a]	<1.097[a]	<0.040[a]
2	2.23	10	0	2.23	0	0.000	0.000	0.000
3	2.25	10	0	2.25	0	0.000	0.000	0.000
4	2.27	10	0	2.27	0	0.000	0.000	0.000
5	2.23	10	1	2.03	0	<0.493[a]	<1.099[a]	<0.041[a]

[a]In mice with contamination detected in one or more of the plates while the rest of the plates had no MTB colonies, one colony was assigned as the total CFU count of uncontaminated plates to calculate CFU/lung.

TABLE S6 Calculation of Log_{10} CFU/lung in the relapse-preventing study.

This table contains data from individual mouse with contaminated plates that prevented a direct summation of the total CFU counts. If contamination was detected in one or more of the plates while the rest contained countable colonies, the contaminated plate was excluded from the calculation. In this situation, CFUs were summed from countable plates, then divided by the total volume spread on these countable plates to obtain CFU/mL, which was then timed with the total initial plated volume and the dilution factor to obtain the CFU/lung for each mouse. In these mice, if all plates had no CFU, they were considered culture negative (CFU = 0). However, if contamination was detected in one or more of the plates while the rest of the plates had no MTB colonies, one colony was assigned as the total CFU count of uncontaminated plates. The CFU/lung was then calculated as follows: 1 CFU divided by the total volume of lung homogenates plated on the uncontaminated plates, which was then timed with the total volume of the original lung homogenates. This value was considered as the detection limit of our CFU counting methodology, as described in the Method section. Values obtained using this detection limit methodology have a prefix of <. The mean and SD data obtained in this table are shown in Table 4.

Animal ID	(A) Total volume of lung homogenate (mL)	Number of 7H11 plates	Number of contaminated plates	(B) Sum of lung homogenate spread on uncontaminated plates (mL)	(C) Number of detected colony	CFU/mL (C)÷(B)	CFU/lung (C)÷(B)×(A)	Log_{10} CFU/lung
Group: RHZE/RH-12w Mean 0.134, SD 0.084								
1	2.24	7	2	1.64	0	<0.610[a]	<1.366[a]	<0.135[a]
2	2.21	7	3	1.31	0	<0.763[a]	<1.687[a]	<0.227[a]
3	2.23	7	2	1.50	0	<0.667[a]	<1.487[a]	<0.172[a]
4	2.23	7	2	1.63	0	<0.613[a]	<1.368[a]	<0.136[a]
5	2.23	7	0	2.23	0	0.000	0.000	0.000
Group: RHZE/RH-14w Mean 0.396, SD 0.445								
1	2.23	7	1	1.80	0	<0.556[a]	<1.364[a]	<0.093[a]
2	2.25	7	2	1.65	0	<0.606[a]	<1.364[a]	<0.135[a]
3	2.26	7	2	1.50	6	4.000	9.040	0.956
4	2.26	7	1	1.80	5	2.778	6.278	0.798
5	2.24	7	0	2.24	1	0.446	1.000	0.000

Group: DCMB-10w Mean 0.038, SD 0.034								
1	2.22	7	1	1.92	0	<0.521[a]	<1.156[a]	<0.063[a]
2	2.24	7	1	1.94	0	<0.515[a]	<1.155[a]	<0.062[a]
3	2.28	7	0	2.28	0	0.000	0.000	0.000
4	2.23	7	0	2.23	0	0.000	0.000	0.000
5	2.22	7	1	1.92	0	<0.521[a]	<1.156[a]	<0.063[a]
Group: DCMB-12w Mean 0.086, SD 0.099								
1	2.22	7	2	1.62	0	<0.617[a]	<1.370[a]	<0.137 [a]
2	2.24	7	1	1.94	0	<0.515[a]	<1.155[a]	<0.062[a]
3	2.17	7	3	1.27	0	<0.787[a]	<1.709[a]	<0.233[a]
4	2.30	7	0	2.30	0	0.000	0.000	0.000
5	2.23	7	0	2.23	0	0.000	0.000	0.000
Group: DCMB-14w Mean 0.044, SD 0.098								
1	2.24	7	0	2.24	0	0.000	0.000	0.000
2	2.22	7	0	2.22	0	0.000	0.000	0.000
3	2.27	7	3	1.37	0	<0.730[a]	<1.657[a]	<0.219[a]
4	2.25	7	0	2.25	0	0.000	0.000	0.000
5	2.22	7	0	2.22	0	0.000	0.000	0.000

[a]In mice with contamination detected in one or more of the plates while the rest of the plates had no MTB colonies, one colony was assigned as the total CFU count of uncontaminated plates to calculate CFU/lung.

Reference

1. Andries K, Villellas C, Coeck N, Thys K, Gevers T, Vranckx L, Lounis N, de Jong BC, Koul A. 2014. Acquired resistance of Mycobacterium tuberculosis to bedaquiline. PloS One 9:e102135.
